# Demographic, risk behaviour and personal network variables associated with prevalent hepatitis C, hepatitis B, and HIV infection in injection drug users in Winnipeg, Canada

**DOI:** 10.1186/1471-2458-6-229

**Published:** 2006-09-13

**Authors:** John L Wylie, Lena Shah, Ann M Jolly

**Affiliations:** 1 Department of Medical Microbiology, University of Manitoba, Winnipeg, Manitoba, Canada; 2 Department of Community Health Sciences, University of Manitoba, Winnipeg, Manitoba, Canada; 3 Cadham Provincial Laboratory, Manitoba Health, Winnipeg, Manitoba, Canada; 4 The Centre for Infectious Disease Prevention and Control, Population and Public Health Branch, Health Canada, Ottawa, Ontario, Canada; 5 Department of Epidemiology and Community Medicine, University of Ottawa, Ottawa, Ontario, Canada

## Abstract

**Background:**

Previous studies have used social network variables to improve our understanding of HIV transmission. Similar analytic approaches have not been undertaken for hepatitis C (HCV) or B (HBV), nor used to conduct comparative studies on these pathogens within a single setting.

**Methods:**

A cross-sectional survey consisting of a questionnaire and blood sample was conducted on injection drug users in Winnipeg between December 2003 and September 2004. Logistic regression analyses were used to correlate respondent and personal network data with HCV, HBV and HIV prevalence.

**Results:**

At the multivariate level, pathogen prevalence was correlated with both respondent and IDU risk network variables. Pathogen transmission was associated with several distinct types of high-risk networks formed around specific venues (shooting galleries, hotels) or within users who are linked by their drug use preferences. Smaller, isolated pockets of IDUs also appear to exist within the larger population where behavioural patterns pose a lesser risk, unless or until, a given pathogen enters those networks.

**Conclusion:**

The findings suggest that consideration of both respondent and personal network variables can assist in understanding the transmission patterns of HCV, HBV, and HIV. It is important to assess these effects for multiple pathogens within one setting as the associations identified and the direction of those associations can differ between pathogens.

## Background

The likelihood of exposure to bloodborne pathogens is a multifactoral process primarily dependent on the risk behaviours an individual practices and the likelihood that a susceptible individual will come into contact with an infected individual (thus increasing or decreasing the risk actually associated with a risk behaviour). The interactions that bring susceptible and infected individuals into contact with each other occur within the context of social networks, the overall structure of which can also affect the rate of pathogen spread [1,2]. Chance events also play a role as a social network may contain a group of individuals whose behaviours favour transmission, but until such time as a bloodborne pathogen enters that network, they face no risk of acquiring that pathogen.

Individual risk behaviours for injection drug users (IDU) include those directly associated with transmission, such as the use of syringes previously used by another IDU or those behaviours which can act as markers of the above types of behaviours. Examples of risk markers that are positively associated with disease prevalence include drug scene roles, such as dealing drugs or injecting others as a service (street or hit doctors) [3-6]. Other behaviours, such as obtaining clean needles from questionable sources such as drug dealers, shooting gallery owners, or on the street, can also act as a marker of an increased probability of using a contaminated needle, as doctoring of used needles to make them appear new has been reported [7].

Social network analysis is a technique which measures and analyzes the interactions that occur between people and its application can enhance an otherwise overly simplistic interpretation of individual risk behaviours. In Brooklyn, injecting with a used syringe was associated with HIV only if syringes were being shared with someone who was 10 or more years older than the interviewed subject, or was a daily injector [8]. By incorporating network variables indicative of a higher probability of coming into contact with an infected individual, a better indication is obtained of who is at risk of infection and what actually constitutes a high-risk behaviour.

Subsequent research on IDU and their network characteristics have identified other network variables associated with transmission risk. High-risk injection practices have been linked to network characteristics such as the number of network members [9,10]; the presence of family members or spouses within the network [4,11]; higher network density [10]; the setting where injection takes place [10]; turnover of network members [12]; and the pooling of financial resources within networks for the purpose of obtaining drugs [9]. Racial/ethnic differences in HIV prevalence have also been at least partially explained by taking into account the differing network characteristics of different ethnic groups [13].

Some individual behaviours or characteristics may also be proxy markers of network behaviours. The type of drug an IDU chooses to inject can be measured as a characteristic of the individual injector and itself can influence risk, as some drugs, like cocaine, are prepared at room temperature and hence are more conducive to pathogen survival [14]. In addition to these more risky drug-specific practices, IDU may form networks based on drug type, which mark the broader social network within which an individual is a member [15,16]. Therefore, network members are more likely to come into contact with whichever pathogen(s) happen to be circulating within that network. Similarly, moving to a new city within the past year can also be an indicator of higher risk as individuals create a social bond through the sharing of drug equipment to try and establish themselves in new networks [12,17].

We have recently described networks centered on downtown hotels in the core area of Winnipeg (manuscript in press), which demonstrate that the setting where an individual injects drugs can also act as a proxy marker of a social network in which some of the members may not know each other, but are nonetheless linked by a common venue. Within these venues accidental or intentional use of contaminated syringes is more likely. Injection at a shooting gallery is another example of a geographic place-based network that has also frequently been linked to increased risk of infection by a bloodborne pathogen [6,18,19].

The majority of the network research described above has focused on HIV and relatively little on hepatitis B and C (HBV and HCV, respectively). Comparative studies of network variables associated with the transmission of HIV, HCV and HBV within the same setting have also not been conducted. Our goal was to determine whether respondent and network variables (including some proxy markers of networks as described above) are associated with the prevalence of HCV, HBV, and HIV in our setting and to what extent similar or dissimilar patterns emerge for the different pathogens.

## Methods

### Study setting

A cross-sectional survey of IDU in Winnipeg, Manitoba, Canada (pop. 675,000) from December, 2003 to September, 2004 was conducted. Potential study recruits were invited to participate through advertisements posted at local community health centres, and meeting places such as laundromats, which had been suggested by key informants within the target population. Word-of-mouth advertising also occurred in the community as the study progressed.

Eligibility criteria were self-reported use of illicit injection drugs in the 6 month period prior to interview and age 15 years or more. Potential participants made telephone contact with the study nurse, who administered all the surveys and collected a blood specimen. An honorarium of $40 Cdn was provided to all study participants, regardless of whether they completed all parts of the study. Participants provided written or oral consent. The study design was approved by the Health Research Ethics Board of the University of Manitoba and the Winnipeg Regional Health Authority Research Review Committee.

### Survey instrument

The questionnaire was divided into three sections. Section 1 consisted of questions based on the respondent's own demographic or behavioural characteristics (hereafter referred to as respondent variables).

Section 2 elicited information on the study participant's egocentric network. Study participants were asked to think back over the last 30 days about the people with whom they had had more than casual contact. Prompts included friends, relatives, or other individuals to whom they feel close, and people with whom they had used drugs, had sex, resided, or hung out. Using initials or other anonymous identifiers, participants were asked to list a maximum of 20 members of their egocentric network (referred to as egocentric variables).

Section 3 elicited information on the respondent's IDU risk network. In this section, a series of detailed questions were asked about each of the individuals within the egocentric network that had been identified by the respondent as IDU (to a maximum of 5 IDU). If more than 5 IDU were listed in the egocentric network, only the first 5 IDU that were listed in the egocentric network were chosen. Respondents then were asked questions about each member of his or her IDU network (e.g. have you ever used a syringe after [person] used it first?). Respondents could then provide different responses for each of the IDU within their risk network (these variables are referred to IDU risk network variables).

### Measures

#### Respondent sociodemographic variables

Demographic variables included age, age of initiation of injection drug use (analyzed as number of years a respondent had injected drugs; referred to as "years of ID use"), gender, level of education completed, ethnicity and whether respondents had moved to Winnipeg in the past year.

Preliminary examination of the data indicated age and years of ID use were correlated; years of ID use only was chosen for use in this analysis. The relationship between years of ID use and serostatus was nonlinear (examined with Lowess smoothed plots [20]). In general, during the first years of drug use, there was a rapid increase in serostatus, followed by a more gradual increase until approximately year 20 after which it leveled off (data not shown). These breakpoints approximated the quartile distribution of years of ID use, therefore this variable was recoded to quartiles.

Due to small sample size, transgender persons (n = 5) and those reporting an ethnicity other than Caucasian or Aboriginal (n = 10) were excluded from analysis. Educational level was coded to two categories: individuals who dropped out of school and those who graduated grade 12 or were currently in school. A recent move to Winnipeg was categorized as those respondents who had moved to Winnipeg in the past year.

### Respondent drug-related behaviours

Respondents were asked questions about their drug-related behaviours, which generally covered the 6 month period prior to interview, but exceptions are noted. Three variables represented use of the three most common drugs: cocaine, a combination of talwin and ritalin (talwin/ritalin) and morphine. A fourth variable for heroin injection (a relatively rare drug in Winnipeg) was included for means of comparison with other studies and because anecdotal evidence suggested that heroin users may be somewhat isolated from other types of users in Winnipeg forming a distinct network with distinct properties. Frequency of drug use was coded as daily vs. non-daily use.

Two binary variables contained data on injection at hotels or shooting galleries. Data on whether respondents had used anyone else's used syringes (ever and during the last 6 months); other related drug-preparation equipment (last 6 months; prompts included cooker, rinse water, or cotton); engaged in drug transfer behaviours (prompts included backloading, frontloading or piggybacking) from another user's syringe to their own (last 6 months) were evaluated. Univariate analysis showed that only ever-use of previously used syringes was significant and is the only one of the above variables presented in this paper (e.g. the univariate OR relating HBV serostatus and use of other's equipment was 0.98 [0.62, 1.55] while the univariate OR for HBV serostatus and engaging in drug transfer behaviours was 0.94 [0.53, 1.67]). Injecting someone as a service was defined as someone who reported that he or she had ever received any money, drugs, or other goods in exchange for injecting someone with drugs.

Obtaining needles from questionable sources such drug dealers, shooting gallery owners, or syringes found on the street was evaluated separately from those who obtained needles from friends or family members, both in the last six months. Both were constructed as binary variables.

While sexual behaviours are linked to HBV and HIV transmission, they are not considered an important route of transmission for HCV, but still indicate certain roles or networks which may be relevant and predictive of risk. We included six binary variables denoting opposite-sex or same-sex sex partnerships in the last six months of the following type; regular (someone with whom the participant is emotionally involved with), casual (someone with whom the participant has had sex with only a few times and has no emotional involvement with) or client (someone who has given the participant money or goods in exchange for sex).

### Egocentric network variables

We created two continuous variables from the total number of people on the network list whom the participant identified as IDU and the total number of people on the list who were identified as both IDU and family members.

### IDU risk network variables

A series of variables were based on the interactions which participants had with individual IDU within their egocentric network (maximum five IDU as noted above). For example. a participant could indicate that he or she saw daily anywhere from none to five of the IDU on their IDU risk network list. To avoid small cell sizes, data were collapsed to either three (0, 1, or 2–5 IDU) or four (0, 1, 2, or 3–5 IDU) dummy variable categories. Twelve interactions with other IDU were considered, including how many IDU risk members 1) the participant sees on a daily basis, 2) inject daily, 3) have been IDU for more than 5 years, 4) have injected at a hotel, 5), have injected at a shooting gallery, 6) the participant has pooled resources with to obtain drugs, 7) have used a syringe before the participant has used it, 8) have used other drug preparation equipment before the participant has used it, 9) have initiated the respondent to drug use, 10) have shown the respondent how to inject drugs, 11) have injected the respondent with drugs, 12) use talwin/ritalin, 13) cocaine, or 14) morphine (heroin was not included here as only 18 respondents knew any heroin users). Variables 7 and 8 are similar to respondent variables regarding the sharing of syringes and equipment (across all partners), however, we felt asking the questions within a network context may result in better recall of these events. Similarly, given the importance of drug type and infection risk, we included variables 12–14.

### Diagnostic testing

Venous blood specimens were collected for HCV, HBV and HIV serological testing at Cadham Provincial Laboratory (Winnipeg, MB). Specimens were screened for HCV and HIV with AxSYM HCV (Abbott, Mississauga, ON) and AxSYM HIV1/2 gO (Abbott, Mississagua, ON), respectively. Presumptive positives were confirmed for HCV with Chiron HCV 3.0 RIBA (Ortho-Clinical Diagnostics, Markham, ON). Presumptive HIV positive specimens were confirmed by western blot (BioRad, Montreal, QC). HBV cases were considered to be those specimens positive for antibodies against HBV core protein (IMX HBV core IgG, Abbott, Mississauga, ON).

### Statistical analysis

Logistic regression was used to analyze the three binary dependent variables (HCV, HBV, and HIV serostatus). The multicollinearity of independent variables was first assessed with a correlation matrix [21]. As noted above, age and years of ID use were correlated and years of ID use was chosen for analysis. The only other two variables that were clearly correlated were respondent's use of talwin/ritalin and use of talwin/ritalin by IDU risk network members. Given that we were interested in comparing variables from an individual *vs*. network perspective, we did not want to choose one of these variables over the other, nor combine them into one variable. Therefore, for each pathogen, we constructed two models where either one or the other of the above variables was used. In all cases, the inclusion of one of these variables over the other had no major effect on the other independent variables that remained in the final models or on the final odds ratios (data not shown). We present only the models using IDU risk network use of talwin/ritalin as it illustrated a trend toward increasing prevalence for some pathogens as the number of talwin/ritalin users within the risk network increased. All remaining variables that were entered into multivariable analysis were also checked for multicollinearity using NCSS (Kaysville, UT). Variance inflation factor and tolerance statistics were within acceptable limits for all variables.

Univariate analyses were first completed and all variables with p values of 0.2 or less were considered for inclusion in multivariable analyses. Logistic regression model building procedures followed those described by Hosmer and Lemeshow [20]. Multivariable analysis was first conducted by entering respondent variables into a regression model. The effect of removing individual variables was assessed through the likelihood ratio test. After final selection of respondent variables, egocentric and IDU risk network variables were entered and the effect of removing each respondent and network variable was again tested with the likelihood ratio test. After creating preliminary main effects models, interactions were assessed between the respondent variable, injection with a used syringe, and all other variables in the model. These interactions were assessed to determine whether any of the variables in the model may affect the risk associated with syringe sharing. All statistical analysis was done using STATA version 8 (Stata corporation, College Station, TX). Diagnostics for the final models were performed using the Hosmer-Lemeshow goodness of fit statistic and the area under the receiver operating characteristic curve using the lfit and lroc commands in STATA [20].

## Results

A total of 435 IDU were enrolled in the study. Some IDU were unable or unwilling to provide a blood specimen. Additionally, five individuals who self-identified as transgender and 10 individuals who self-identified as belonging to ethnic groups other than Caucasian or Aboriginal, were excluded from the analysis. For the remaining 369 respondents, summary statistics for the egocentric network are as follows: mean number of members, 8.8; median, 8; range, 1–20, lower quartile, 5; upper quartile, 11; SD, 4.6. Given that the IDU risk network was truncated at 5 IDU, we also report these summary statistics for the total number of people within the egocentric network identified as IDU: mean number of IDU in the egocentric network, 3.5; median, 3; range, 0–20; lower quartile, 1; upper quartile, 5. In total, 17% of respondents (66/369) reported more than 5 IDU in their egocentric network. The lack of data on these individuals is discussed further in the limitations section of this paper. Of the 369 respondents considered for analysis, several indeterminate results were identified for each pathogen therefore, the final sample sizes for HCV, HBV, and HIV were 365, 364, and 360, respectively.

### Correlation between HCV, HBV, and HIV

To assist in data interpretation the correlation between HCV, HBV, and HIV were determined. Pairwise correlation values for HCV/HBV serostatus was 0.001; HCV/HIV, -0.011; and HBV/HIV, -0.011. No apparent correlation between the three pathogens was evident.

### Univariate analyses

The univariate analyses for these 3 pathogens are shown in tables 1, 2, 3. In univariate analyses, the subset of variables associated with serostatus and the direction of that association was similar for the three pathogens. Respondent variables positively associated with serostatus for all three pathogens were: years of ID use, aboriginal ethnicity, injection at a shooting gallery, ever reporting the use of someone's else's used syringe; injecting someone else as a service; and having opposite-sex client partners.

Thirteen variables were associated with both HCV and HBV, but not HIV. Positive associations were found for two respondent variables (having injected at a hotel; injecting talwin/ritalin), two egocentric network variables (total number of IDU; total number of family IDU), and five IDU risk network variables (the number of IDU who inject daily, the number of IDU who have been injecting for more than 5 years, the number of IDU who have injected talwin/ritalin, the number of IDU who have injected at a hotel, and the number of IDU who have used a syringe before the respondent used it). Negative associations were found for three respondent variables (moved to Winnipeg in the previous year; injection of heroin and daily injection) and one IDU risk network variable (number of IDU who initiated the respondent to injection drug use).

Two respondent variables were associated with HCV and HIV, but not HBV. A negative association was found for obtaining clean syringes from friends. Reporting same-sex regular sex partners was negatively associated with HCV and positively associated with HIV.

Ten variables were associated with only one of the three pathogens. Positive associations were found between HCV and one respondent variable (obtains clean syringes from questionable sources); between HBV and four IDU risk network variables (the number of IDU in the risk network who have injected at a shooting gallery; the number of IDU with whom the respondent has pooled resources; the number of IDU who have injected the respondent and the number of IDU who have injected cocaine); between HIV and two respondent variables (injection of cocaine and same-sex client sex partners). Negative associations were found between HBV and reporting same-sex casual sex partners and opposite-sex casual partners and between HCV and the number of IDU in the risk network who have shown the respondent how to inject drugs.

### Multivariate analyses

In multivariate analysis, prior to assessing interactions, HCV was positively associated with respondent's age of initiation to injection drug use, reported injection with a syringe used by another IDU, injection at a shooting gallery, injecting someone as a service, and the number of IDU in the risk network who inject talwin/ritalin (table 4). Negative associations for this pathogen were found for the respondent obtaining syringes from friends and the number of IDU in the risk network who had shown the respondent how to inject drugs.

For the model described above, interactions were assessed between the respondent variable, injection with a used syringe, and the remaining variables in the model. An interaction was noted between injection with used syringes and the number of IDU network members who inject talwin/ritalin and the number of IDU network members who had shown the respondent how to inject drugs (table 5). For respondents who did not associate with other talwin/ritalin users, injecting with syringes previously used by other IDUs was positively associated with HCV prevalence. This correlation was absent among those IDU linked to talwin and ritalin, such that their HCV prevalence was high regardless of whether or not they reported injecting with used syringes. Similarly, for those IDU who were in a risk network where someone had shown them how to inject drugs, HCV prevalence was relatively low and not associated with injection with a used syringe. After adjusting for these interactions, two respondent variables, obtaining needles from friends and injecting someone as a service, were no longer significant.

HBV was positively associated with three respondent variables: years of ID use, injection with a used syringe, and injection at a shooting gallery and two IDU risk network variables: the number of IDU in the risk network who injected talwin/ritalin, and the number who had injected at a hotel (table 6). The latter variable was significant only if the respondent identified 2 or more IDU in the risk network with this characteristic. HBV was negatively associated with reporting opposite-sex casual sex partners; moving to Winnipeg in the past year and the number of IDU in the risk network who had initiated the respondent to injection drug use. As above, this variable was only significant if 2 or more IDU in the risk network were reported as having initiated the respondent to injection drug use.

HIV was positively associated with four respondent variables; injection with used syringes, injection at a shooting gallery, same-sex client sex partners and aboriginal ethnicity (table 7).

## Discussion

Our two primary goals in this investigation were to determine which respondent and/or network variables were associated with HCV, HBV, and HIV in our setting and to compare and contrast these variables for the different pathogens. Only two respondent variables were positively associated with all three pathogens: injection with syringes previously used by another IDU and injection at a shooting gallery. The former represents a direct route for parenteral transmission of blood borne pathogens and is clearly a common factor for transmission of all three of the pathogens examined in our study area. The latter, given the high-risk activities characteristic of this type of venue [6,18,19], would be associated with an increased likelihood of exposure through purposeful or accidental use of contaminated equipment. Although this variable is a marker for high-risk behaviour on the part of individuals, it is also a proxy marker for membership in high risk, largely anonymous networks of IDU. Networks form around specific places where geographic place is the common connecting tie between individuals [22-24]. Including place in network analysis adds explanatory power for understanding disease transmission regardless of the infection. Here shooting galleries are not simply high risk environments for individual users, but, by acting as repositories for contaminated equipment, they create bridging opportunities for pathogens between individuals who may not know each other and who may never otherwise meet.

Like shooting galleries, certain hotels in Winnipeg are also important venues for injection and favor formation of place-based networks of IDU. The likelihood of HBV infection increased proportional to the number of IDU the respondent knew who had injected at a hotel. Association with HBV, but not with HCV or HIV, could result from HBV entering the hotel network(s) earlier than HCV and HIV, resulting in more extensive spread. Alternatively, there may be specific behaviours associated with this group of IDU that favour specific transmission of this pathogen (e.g. hotel rooms, as opposed to shooting galleries, may offer more privacy and a greater likelihood of engaging in sexual activities, thus favouring HBV transmission over HCV). Regardless of the causal factors involved, like others [8,25], we found that incorporating interactions with risk network members provided a clearer understanding of what actually constitutes high-risk behaviour.

Respondents' injection of talwin/ritalin and their use by IDU network members was a marker for HCV and HBV. The type of drug used can loosely demarcate distinct networks of IDU [15,16]. Within these networks specific behaviours may favour transmission (e.g. the room temperature production of cocaine *vs*. the need to heat some other types of drugs [14]). To our knowledge, extensive use of talwin/ritalin is relatively rare outside of the Canadian provinces of Manitoba and Saskatchewan. It is notable that in our analysis, injection with used syringes interacted with talwin/ritalin such that the prevalence of HCV was high amongst talwin/ritalin users regardless of whether or not they reported injection with used syringes. Like cocaine, talwin/ritalin is typically prepared at room temperature and frequently involves communal use of the prepared drug and filters, which may account for the high prevalence of HCV regardless of syringe sharing practices (typically pills are crushed in room temperature water; IDU then use a common filter to draw drug into their own respective syringes). It is also notable, that HIV does not show a positive association with talwin/ritalin in contrast to HCV and HBV. As above, this may reflect a different temporal framework for pathogen entry to the network of talwin/ritalin users. If this is the case, the same behaviours which facilitated spread of HBV and HCV may ultimately lead to high HIV prevalence in this group.

Two IDU risk network variables were negatively associated with either HCV or HBV. For HBV, there was a negative association with the number of IDU in the respondent's risk network who had initiated them to injection drug use, while for HCV there was a negative association if the respondent reported that there were IDU in their risk network who had shown them how to inject drugs. For HCV, there was also a weak interaction between the number of IDU members who had shown the respondent how to shoot drugs and syringe sharing, such that syringe sharing was no longer positively associated with prevalence. In general, these network variables may mark small, relatively closed networks of IDU who may share equipment and syringes, but rarely admit new members. Low network turnover has been associated with lower risk [12] as opportunities for entry of pathogens to these networks (via infected individuals) would be correspondingly reduced. An area worthy of research would be whether the lower prevalence of infection is a chance byproduct of the social interactions within these networks or whether the individuals within closed networks are actively and knowingly attempting to isolate themselves from infection risk.

The greater likelihood of HIV and HBV being transmitted through sexual contact was consistent with sex partner-related variables being retained in the final models for these two pathogens. However, while a positive correlation for HIV occurred with same-sex client partners, the directionality for HBV and opposite-sex casual partners was opposite. Although the direction of this relationship appears counter-intuitive, it may reflect a greater likelihood to engage in safer sex harm reduction activities with sex partners of this type. This question could be addressed in more detailed research on sexual contact behaviours in this population.

Aboriginal ethnicity and a recent move to Winnipeg were associated with HIV and HBV, respectively. HIV was the only pathogen where ethnicity remained as an independent predictor of risk in the final model. Research in other cities have identified specific social network interactions that can account for ethnic differences in disease prevalence [13]. Further research would be necessary within our study population to identify what risk behaviour(s) may be associated with HIV infection in the aboriginal population in Manitoba that may not have been captured by our survey. The relatively small number of HIV positive individuals in our dataset also may have limited the analytic potential for this pathogen in regression analysis. A recent move to Winnipeg was associated with a lesser likelihood of HBV positivity. This pattern may reflect a lower prevalence of HBV and/or earlier or more extensive deployment of vaccination programs in the provinces from which these IDU originated (e.g. the province of British Columbia began vaccinating approximately 6 years prior to Manitoba).

A number of limitations for the study should be noted. First, the study was cross-sectional and hence does not provide any data on trends or whether currently observed behaviours were a result of, rather than the cause of, an infection by one of the pathogens studied. Second, the data were egocentric and hence no independent validation of the behaviours of IDU in the risk network was possible. Third, network members were elicited based on contact in the previous 30 days, but questions regarding individual behaviours and interactions with network members were typically collected for the previous 6 months (or longer periods for some variables such as "ever-use of someone else's syringe"). Time frames are relevant, given the noted lack of correlation between some known risk behaviours and serostatus in our analysis (see paragraph below). Any comparisons of our results with those from other areas would need to take note of the time frames used. Fourth, the egocentric and IDU risk networks were truncated at 20 and 5 people, respectively. The implications of this data truncation are greatest for the IDU risk network variables as they formed a large part of the analysis. Overall, 17% of respondents reported more than 5 IDU in their egocentric network. We felt that full data collection on all IDU in the egocentric network was impractical in terms of time, complexity of data collection and accurate recall by respondents, however, it should be noted that this additional data could have revealed additional patterns not apparent in our results.

Finally, some limitations regarding specific variables should be noted. First, data on sharing of drug-preparation equipment and transfer of prepared drugs between syringes was available for only the six months prior to interview. In contrast, sharing of used syringes was based both on six-month and "ever" data. Only the latter was a predictor of risk, therefore, if more extensive data had been collected for equipment sharing and drug transfers, they may also have been significant predictors of risk of infection for some, or all, of the pathogens studied. Second, only a limited number of sexual behaviour questions were included in the study and no specific questions on type of sex were asked. Collection of data on anal sex, in particular, could have revealed other correlations not evident with the dataset available. Third, only "injecting someone else as a service" was analyzed; "injecting someone else as a favor" or "receiving injections from others" can be considered distinct traits and could also be related to the common use of syringes and pathogen transmission.

## Conclusion

Our findings reinforce the clear link between respondent behaviours (e.g. injection with someone else's used syringe) and risk of infection. However, they also highlight the importance of defining the types of personal networks that form within an area and identifying the different patterns of transmission of pathogens within those networks. In our setting, pathogen transmission is associated with loosely defined, sometimes anonymous, high-risk networks formed around specific venues (shooting galleries, hotels) or between users who are linked by their drug use preferences. Smaller, somewhat isolated pockets of IDUs appear to exist within the larger population where behavioural patterns pose a lesser risk, unless, or until, a given pathogen enters those networks. Temporal differences with respect to pathogen introduction to the different networks likely influences which specific behaviours appear as protective or as high-risk, highlighting the importance of verifying the extent to which risk behaviours or markers identified as important in one setting or to one pathogen apply to other settings or pathogens. The lack of any apparent correlation between the prevalent infections for the three pathogens, noted in the results section, reinforces the need to identify the different social and temporal patterns associated with the various pathogens that may be circulating within a population. Both individual- and network-level concepts assist in characterizing and comparing the transmission of HCV and HBV, as well as, HIV.

A potential hierarchy of risk emerges, defined by space and time. Individuals may have some independence in their decisions to use drugs or not, or share syringes or not. However, the broader personal network in which they find themselves, whether defined by living near a hotel where drug use is common or by their ethnic or age group, is no less important in contributing to their potential for initiation of specific behaviours or exposure to infectious agents. The smaller, more specific social interactions with their IDU risk network forms an additional step in a progression which may determine an individual's choice of drug; their norms, rituals and/or practices of injection; and their likelihood of exposure to infected individuals. Both individual- and network-level concepts assist in characterizing and comparing the transmission of HCV, HBV, and HIV and determining the underlying patterns that drive the social connections between individuals that may favour or hinder transmission of specific pathogens.

## Competing interests

The author(s) declare that they have no competing interests.

## Authors' contributions

JW and AJ conceived and designed the study. JW oversaw implementation of the study in Winnipeg and drafted the manuscript. LS prepared the database for analysis and both JW and LS performed the statistical analysis. AJ and LS assisted with manuscript editing and all authors read and approved the final manuscript.

## Pre-publication history

The pre-publication history for this paper can be accessed here:



## Figures and Tables

**Table 1:** Odds ratios and 95% confidence intervals for univariate analysis of HCV serostatus.

Variable name	HCV negative (n = 167) (%)	HCV positive (n = 198) (%)	OR (95% CI)	p value
***Respondent variables***				
**Years of injection drug use (quartiles)**				
0–6	81 (48.5)	20 (10.1)	1.0	
7–13	46 (27.5)	51 (25.8)	4.5 (2.4–8.4)	<0.001
14–21	19 (11.4)	61 (30.8)	13.0 (6.4–26.5)	<0.001
22–39	21 (12.6)	66 (33.3)	12.7 (6.4–25.5)	<0.001
**Gender**				
Male	96 (57.5)	113 (57.1)	1.0	
Female	71 (42.5)	85 (42.9)	1.0 (0.7–1.5)	0.937
**Education level**				
< grade 12	116 (69.5)	142 (71.7)	1.0	
> grade 12	51 (30.5)	56 (28.3)	0.9 (0.6–1.4)	0.637
**Ethnicity**				
Caucasian	71 (42.5)	57 (28.8)	1.0	
Aboriginal	96 (57.5)	141 (71.2)	1.8 (1.2–2.8)	0.006
**Moved to Winnipeg in past year**				
No	112 (67.1)	153 (77.3)	1.0	
Yes	55 (32.9)	45 (22.7)	0.6 (0.4–1.0)	0.030
**Deals drugs**				
No	85 (50.9)	107 (54.0)	1.0	
Yes	82 (49.1)	91 (46.0)	0.9 (0.6–1.3)	0.549
**Cocaine use**				
No	62 (37.1)	69 (34.9)	1.0	
Yes	105 (62.9)	129 (65.1)	1.1 (0.7–1.7)	0.651
**Talwin/ritalin use**				
No	134 (80.2)	113 (57.1)	1.0	
Yes	33 (19.8)	85 (42.9)	3.1 (1.9–4.9)	<0.001
**Morphine use**				
No	110 (65.9)	121 (61.1)	1.0	
Yes	57 (34.1)	77 (38.9)	1.2 (0.8–1.9)	0.347
**Heroin use**				
No	145 (86.8)	184 (92.9)	1.0	
Yes	22 (13.2)	14 (7.1)	0.5 (0.2–1.0)	0.052
**Injects daily**				
No	20 (12.0)	43 (21.7)	1.0	
Yes	147 (88.0)	155 (78.3)	0.5 (0.3–0.9)	0.013
**Injected at a hotel**				
No	109 (65.3)	107 (54.0)	1.0	
Yes	58 (34.7)	91 (46.0)	1.6 (1.0–2.4)	0.029
**Injected at a shooting gallery**				
No	153 (91.6)	156 (78.8)	1.0	
Yes	14 (8.4)	42 (21.2)	2.9 (1.5–5.6)	<0.001
**Ever used someone else's syringe**				
No	90 (53.9)	49 (24.8)	1.0	
Yes	77 (46.1)	149 (75.3)	3.6 (2.3–5.5)	<0.001
**Injected someone else as a service**				
No	144 (86.2)	125 (63.1)	1.0	
Yes	23 (13.8)	73 (36.9)	3.7 (2.2–6.2)	<0.001
**Obtains syringes from friends**				
No	50 (29.9)	73 (36.9)	1.0	
Yes	117 (70.1)	125 (63.1)	0.7 (0.5–1.1)	0.162
**Obtains syringes from questionable sources**				
No	135 (80.8)	144 (72.7)	1.0	
Yes	32 (19.2)	54 (27.3)	1.6 (1.0–2.6)	0.067
**Opposite-sex regular partners**				
No	51 (30.5)	65 (31.8)	1.0	
Yes	116 (69.5)	163 (68.2)	0.9 (0.6–1.4)	0.640
**Opposite-sex casual partners**				
No	101 (60.5)	129 (65.2)	1.0	
Yes	66 (39.5)	69 (34.9)	0.8 (0.5–1.3)	0.357
**Opposite-sex client partners**				
No	151 (90.4)	163 (82.3)	1.0	
Yes	16 (9.6)	35 (17.7)	2.0 (1.1–3.8)	0.024
**Regular same-sex partners**				
No	160 (95.8)	195 (98.5)	1.0	
Yes	7 (4.2)	3 (1.5)	0.4 (0.1–1.4)	0.116
**Casual same-sex partners**				
No	160 (95.8)	191 (96.5)	1.0	
Yes	7 (4.2)	7 (3.5)	0.8 (0.3–2.4)	0.745
**Client same-sex partners**				
No	163 (97.6)	194 (98.0)	1.0	
Yes	4 (2.4)	4 (2.0)	0.8 (0.2–3.4)	0.808
***Egocentric network variables (maximum number of network members = 20)***				
**Total number of IDU in network**				
Median (IQR)	3 (1,5)	3 (1,5)	1.0 (1.0–1.1)	0.194
**Total number of IDU family members in network**				
Median (IQR)	0 (0,0)	0 (0,1)	1.29 (1.1–1.6)	0.10
**Spouse IDU in network**				
No	109 (65.3)	127 (64.1)	1.0	
Yes	58 (34.7)	71 (35.9)	1.1 (0.7–1.6)	0.822
***IDU risk network variables (maximum number of IDU = 5)***				
**Number of IDU respondent sees daily**				
None	54 (32.3)	66 (33.3)	1.0	
1	55 (32.9)	68 (34.3)	1.0 (0.6–1.7)	0.964
2	32 (19.2)	30 (15.2)	0.8 (0.4–1.4)	0.398
3–5	26 (15.6)	34 (17.2)	1.1 (0.6–2.0)	0.832
**Number of IDU in network who inject daily**				
None	104 (62.3)	105 (53.0)	1.0	
1	32 (19.2)	36 (18.2)	1.1 (0.6–1.9)	0.699
2	19 (11.4)	31 (15.7)	1.6 (0.9–3.0)	0.137
3–5	12 (7.2)	26 (13.1)	2.1 (1.0–4.5)	0.042
**Number of IDU in network who have been IDU for >5 years**				
0	101 (60.5)	74 (37.4)	1.0	
1	28 (16.8)	40 (20.2)	1.9 (1.1–3.4)	0.021
2	18 (10.8)	39 (19.7)	3.0 (1.6–5.6)	0.001
3–5	20 (12.0)	45 (22.7)	3.1 (1.7–5.6)	<0.001
**Number of IDU in network who have injected at a hotel**				
0	117 (70.1)	112 (56.6)	1.0	
1	29 (17.4)	39 (19.7)	1.4 (0.8–2.4)	0.222
2–5	21 (12.6)	47 (23.7)	2.3 (1.3–4.2)	0.004
**Number of IDU in network who have injected at a shooting gallery**				
0	134 (80.2)	143 (72.2)	1.0	
1	18 (10.8)	27 (13.6)	1.4 (0.7–2.7)	0.298
2–5	15 (9.0)	28 (14.1)	1.7 (0.9–3.4)	0.102
**Number of IDU in network with whom respondent has pooled money**				
0	51 (30.5)	56 (28.3)	1.0	
1	42 (25.2)	54 (27.3)	1.2 (0.7–2.0)	0.576
2–3	43 (25.8)	55 (27.8)	1.2 (0.7–2.0)	0.587
4–5	31 (18.6)	33 (16.7)	1.0 (0.5–1.8)	0.922
**Number of IDU in network who have used a syringe before the respondent used it**				
0	133 (79.6)	146 (73.7)	1.0	
1	27 (16.2)	32 (16.2)	1.1 (0.6–1.9)	0.790
2–5	7 (4.2)	20 (10.1)	2.6 (1.1–6.4)	0.036
**Number of IDU in network who have used other drug paraphernalia before respondent has used it**.				
0	105 (62.9)	119 (60.1)	1.0	
1	29 (17.4)	31 (16.7)	0.9 (0.5–1.7)	0.841
2–5	33 (19.8)	48 (24.2)	1.3 (0.8–2.1)	0.342
**Number of IDU in network who have initiated respondent to injection drug use**				
0	110 (65.9)	157 (79.3)	1.0	
1	48 (28.8)	35 (17.7)	0.5 (0.3–0.8)	0.008
2–5	9 (5.4)	6 (3.0)	0.5 (0.2–1.4)	0.160
**Number of IDU in network who have shown respondent how to inject drugs**				
0	104 (62.3)	147 (74.2)	1.0	
1	43 (25.8)	39 (19.7)	0.6 (0.4–1.1)	0.083
2–5	20 (12.0)	12 (6.1)	0.4 (0.2–0.9)	0.027
**Number of IDU in network who have injected respondent with drugs**				
0	89 (53.3)	88 (44.4)	1.0	
1	44 (26.4)	54 (27.3)	1.2 (0.8–2.0)	0.392
2–5	34 (20.4)	56 (28.3)	1.7 (1.0–2.8)	0.054
**Number of IDU in network who have used talwin/ritalin**				
0	139 (83.2)	119 (60.1)	1.0	
1–3	17 (10.1)	42 (21.1)	2.9 (1.6–5.3)	<0.001
4–5	11 (6.6)	37 (18.7)	3.9 (1.9–8.0)	<0.001
**Number of IDU in network who have used cocaine**				
0	113 (67.7)	120 (60.6)	1.0	
1	23 (13.8)	36 (18.2)	1.5 (0.8–2.6)	0.192
2–3	17 (10.2)	30 (15.2)	1.7 (0.9–3.2)	0.125
4–5	14 (8.4)	12 (6.1)	0.8 (0.4–1.8)	0.605
**Number of IDU in network who have used morphine**				
0	129 (77.3)	149 (75.3)	1.0	
1	22 (13.2)	31 (15.7)	1.2 (0.7–2.2)	0.513
2–5	16 (9.6)	18 (9.1)	1.0 (0.5–2.0)	0.942

**Table 2:** Odds ratios and 95% confidence intervals for univariate analysis of HBV serostatus.

Variable name	HBV negative (n = 253) (%)	HBV positive (n = 111) (%)	OR (95% CI)	p value
***Respondent variables***				
**Years of injection drug use (quartiles)**				
0–6	97 (38.3)	5 (4.5)	1.0	
7–13	74 (29.3)	23 (20.7)	6.0 (2.2–16.6)	<0.001
14–21	42 (16.6)	36 (32.4)	16.6 (6.1–45.3)	<0.001
22–39	40 (15.8)	47 (42.3)	22.8 (8.4–61.5)	<0.001
**Gender**				
Male	150 (59.3)	60 (54.0)	1.0	
Female	103 (40.7)	51 (46.0)	1.2 (0.8–1.9)	0.353
**Education level**				
< grade 12	178 (70.4)	79 (71.2)	1.0	
> grade 12	75 (29.6)	32 (28.8)	1.0 (0.6–1.3)	0.485
**Ethnicity**				
Caucasian	99 (39.1)	29 (26.1)	1.0	
Aboriginal	154 (60.9)	82 (73.9)	1.8 (1.1–3.0)	0.015
**Mobility**				
In Winnipeg > 1 year	168 (66.4)	97 (87.4)	1.0	
Moved to Winnipeg in past year	85 (33.6)	14 (12.6)	0.3 (0.2–0.5)	<0.001
**Deals drugs**				
No	129 (51.0)	61 (55.0)	1.0	
Yes	124 (49.01	50 (45.1)	0.9 (0.5–1.3)	0.485
**Cocaine use**				
No	85 (33.6)	44 (39.6)	1.0	
Yes	168 (66.4)	67 (60.4)	0.8 (0.5–1.2)	0.27
**Talwin/ritalin use**				
No	190 (75.1)	55 (49.6)	1.0	
Yes	63 (24.9)	56 (50.5)	3.1 (1.9–4.9)	<0.001
**Morphine use**				
No	165 (64.8)	68 (61.3)	1.0	
Yes	89 (35.2)	43 (38.7)	1.2 (0.7–1.8)	0.516
**Heroin use**				
No	222 (87.8)	106 (95.5)	1.0	
Yes	31 (12.3)	5 (4.5)	0.3 (0.1–0.9)	0.015
**Injects daily**				
No	34 (13.4)	26 (23.4)	1.0	
Yes	219 (86.6)	85 (76.6)	0.5 (0.3–0.9)	0.021
**Injected at a hotel**				
No	160 (63.2)	53 (47.8)	1.0	
Yes	93 (36.8)	58 (52.2)	1.9 (1.2–3.0)	0.006
**injected at a shooting gallery**				
No	224 (88.5)	85 (62.2)	1.0	
Yes	29 (11.5)	26 (23.4)	2.4 (1.3–4.2)	0.004
**Ever used someone else's syringe**				
No	114 (45.1)	25 (22.5)	1.0	
Yes	139 (54.9)	86 (77.5)	2.8 (1.7–4.7)	<0.001
**Injected someone else as a service**				
No	204 (80.6)	66 (59.5)	1.0	
Yes	49 (19.4)	45 (40.6)	2.8 (1.7–4.6)	<0.001
**Obtains syringes from friends**				
No	81 (32.0)	41 (16.9)	1.0	
Yes	172 (68.0)	70 (63.1)	0.8 (0.5–1.3)	0.362
**Obtains syringes from questionable sources**				
No	197 (77.9)	81 (73.0)	1.0	
Yes	56 (22.1)	30 (27.0)	1.3 (0.8–2.2)	0.316
**Opposite-sex regular partners**				
No	81 (32.0)	36 (32.4)	1.0	
Yes	172 (68.0)	75 (67.6)	1.0 (0.6–1.6)	0.938
**Opposite-sex casual partners**				
No	149 (58.9)	80 (72.1)	1.0	
Yes	104 (41.1)	31 (27.9)	0.6 (0.3–0.9)	0.015
**Opposite-sex client partners**				
No	223 (88.1)	90 (81.1)	1.0	
Yes	30 (11.9)	21 (18.9)	1.7 (0.9–3.2)	0.081
**Regular same-sex partners**				
No	245 (96.8)	109 (98.2)	1.0	
Yes	8 (3.2)	2 (1.8)	0.6 (0.1–2.7)	0.448
**Casual same-sex partners**				
No	240 (94.9)	109 (98.2)	1.0	
Yes	13 (5.1)	2 (1.8)	0.3 (0.1–1.5)	0.113
**Client same-sex partners**				
No	247 (97.6)	109 (98.2)	1.0	
Yes	6 (2.4)	2 (1.8)	0.8 (0.2–3.8)	0.728
***Egocentric network variables (maximum number of network members = 20)***				
**Total number of IDU in network**				
Median (IQR)	3 (1,4)	3 (2,5)	1.1 (1.0–1.2)	0.001
**Total number of IDU family members in network**				
Median (IQR)	0 (0,1)	0 (0,1)	1.3 (1.0–1.5)	0.019
**Spouse IDU in network**				
No	169 (66.8)	69 (62.2)	1.0	
Yes	84 (33.2)	42 (37.8)	1.2 (0.8–1.9)	0.394
***IDU risk network variables (maximum number of IDU = 5)***				
**Number of IDU respondent sees daily**				
None	83 (32.8)	38 (34.2)	1.0	
1	84 (33.2)	38 (34.2)	1.0 (0.6–1.7)	0.965
2	48 (19.0)	13 (11.7)	0.6 (0.3–1.2)	0.155
3–5	38 (15.0)	22 (19.8)	1.3 (0.7–2.4)	0.479
**Number of IDU in network who inject daily**				
None	160 (63.2)	50 (45.1)	1.0	
1	42 (16.6)	25 (22.5)	1.9 (1.1–3.4)	0.032
2	31 (12.3)	17 (15.3)	1.8 (0.9–3.4)	0.101
3–5	20 (7.9)	19 (17.1)	3.0 (1.5–6.1)	0.002
**Number of IDU in network who have been IDU for >5 years**				
0	142 (56.1)	34 (30.6)	1.0	
1	50 (19.8)	18 (16.2)	1.5 (0.8–2.9)	0.223
2	32 (12.7)	24 (21.6)	3.1 (1.6–6.0)	0.001
3–5	29 (11.5)	35 (31.5)	5.0 (2.7–9.4)	<0.001
**Number of IDU in network who have injected at a hotel**				
0	175 (69.2)	54 (48.7)	1.0	
1	48 (19.0)	20 (18.0)	1.4 (0.7–2.5)	0.330
2–5	30 (11.9)	37 (33.3)	4.0 (2.3–7.1)	<0.001
**Number of IDU in network who have injected at a shooting gallery**				
0	204 (80.6)	74 (66.7)	1.0	
1	30 (11.9)	15 (13.5)	1.4 (0.7–2.7)	0.351
2–5	19 (7.5)	22 (19.8)	3.2 (1.6–6.2)	0.001
**Number of IDU in network with whom respondent has pooled money**				
0	84 (33.2)	23 (20.7)	1.0	
1	62 (24.5)	33 (29.7)	1.9 (1.0–3.6)	0.037
2–3	64 (25.3)	34 (30.6)	1.9 (1.0–3.6)	0.036
4–5	43 (17.0)	21 (18.9)	1.8 (0.9–3.6)	0.103
**Number of IDU in network who have used a syringe before the respondent used it**				
0	191 (75.5)	87 (78.4)	1.0	
1	47 (18.6)	12 (10.8)	0.6 (0.3–1.1)	0.097
2–5	15 (5.9)	12 (10.8)	1.8 (0.8–3.9)	0.168
**Number of IDU in network who have used other drug paraphernalia before respondent has used it**.				
0	155 (61.3)	67 (60.4)	1.0	
1	46 (18.2)	13 (11.8)	0.7 (0.3–1.3)	0.220
2–5	52 (20.6)	31 (27.9)	1.4 (0.8–2.3)	0.234
**Number of IDU in network who have initiated respondent to injection drug use**				
0	177 (70.0)	88 (79.2)	1.0	
1	63 (24.9)	21 (18.9)	0.7 (0.4–1.2)	0.159
2–5	13 (5.1)	2 (1.8)	0.3 (0.1–1.4)	0.128
**Number of IDU in network who have shown respondent how to inject drugs**				
0	172 (68.0)	79 (71.2)	1.0	
1	58 (22.9)	23 (20.7)	0.9 (0.5–1.5)	0.602
2–5	23 (9.1)	9 (8.1)	0.8 (0.4–1.9)	0.700
**Number of IDU in network who have injected respondent with drugs**				
0	133 (52.6)	43 (38.7)	1.0	
1	65 (25.7)	33 (29.7)	1.6 (0.9–2.7)	0.103
2–5	55 (21.7)	35 (31.5)	2.0 (1.1–3.4)	0.015
**Number of IDU in network who have used talwin/ritalin**				
0	197 (77.9	59 (53.2)	1.0	
1–3	31 (12.3)	29 (26.1)	3.1 (1.7–5.6)	<0.001
4–5	25 (9.9)	48 (13.2)	3.1 (1.6–5.8)	0.001
**Number of IDU in network who have used cocaine**				
0	168 (66.4)	64 (57.7)	1.0	
1	39 (15.4)	20 (18.0)	1.3 (0.7–2.5)	0.340
2–3	26 (10.3)	21 (18.9)	2.1 (1.1–4.0)	0.022
4–5	20 (7.9)	26 (7.1)	0.8 (0.3–2.0)	0.625
**Number of IDU in network who have used morphine**				
0	198 (78.3)	80 (72.1)	1.0	
1	34 (13.4)	18 (16.2)	1.3 (0.7–2.5)	0.399
2–5	21 (8.3)	13 (11.7)	1.5 (0.7–3.2)	0.258

**Table 3:** Odds ratios and 95% confidence intervals for univariate analysis of HIV serostatus.

Variable name	HIV negative (n = 334) (%)	HIV positive (n = 26) (%)	OR (95% CI)	p value
***Respondent variables***				
**Years of injection drug use (quartiles)**				
0–6	100 (29.9)	2 (7.7)	1.0	
7–13	83 (24.9)	11 (42.3)	6.6 (1.4–30.7)	0.016
14–21	71 (21.3)	7 (26.9)	4.9 (0.9–24.4)	0.051
22–39	80 (24.0)	6 (23.1)	3.75 (0.7–19.0)	0.111
**Gender**				
Male	193 (57.8)	13 (50.0)	1.0	
Female	141 (42.2)	13 (50.0)	1.4 (0.6–3.0)	0.442
**Education level**				
< grade 12	234 (70.1)	19 (73.1)	1.0	
> grade 12	100 (29.9)	7 (26.9)	0.9 (0.4–2.1)	0.744
**Ethnicity**				
Caucasian	125 (37.4)	3 (11.5)	1.0	
Aboriginal	209 (62.6)	23 (88.5)	4.6 (1.4–15.6)	0.004
**Mobility**				
In Winnipeg > 1 year	244 (73.1)	18 (69.2)	1.0	
Moved to Winnipeg in past year	90 (27.0)	8 (30.8)	1.2 (0.5–2.9)	0.677
**Deals drugs**				
No	175 (52.4)	15 (57.7)	1.0	
Yes	159 (47.6)	11 (42.3)	0.8 (0.4–1.8)	0.601
**Cocaine use**				
No	122 (36.5)	6 (23.1)	1.0	
Yes	212 (63.5)	20 (76.9)	1.9 (0.7–4.9)	0.154
**Talwin/ritalin use**				
No	228 (68.3)	16 (61.5)	1.0	
Yes	106 (31.7)	10 (38.5)	1.3 (0.6–3.1)	0.486
**Morphine use**				
No	209 (62.6)	21 (80.8)	1.0	
Yes	125 (37.4)	5 (19.2)	0.4 (0.1–1.1)	0.051
**Heroin use**				
No	299 (89.5)	26 (100)		
Yes	35 (10.5)	0 (0.00)	Not defined	
**Injects daily**				
No	52 (15.6)	5 (19.2)	1.0	
Yes	282 (84.4)	21 (80.8)	0.8 (0.3–2.1)	0.631
**Injected at a hotel**				
No	198 (59.3)	16 (61.5)	1.0	
Yes	136 (40.7)	10 (38.5)	0.9 (0.4–2.1)	0.821
**injected at a shooting gallery**				
No	287 (85.9)	17 (65.4)	1.0	
Yes	47 (14.1)	9 (34.6)	3.2 (1.4–7.7)	0.012
**Ever used someone else's syringe**				
No	136 (40.7)	2 (7.7)	1.0	
Yes	198 (59.3)	24 (92.3)	8.2 (1.9–35.5)	<0.001
**Injected someone else as a service**				
No	256 (76.7)	13 (50.0)	1.0	
Yes	78 (23.3)	13 (50.0)	3.3 (1.5–7.4)	0.005
**Obtains syringes from friends**				
No	106 (31.7)	13 (50.0)	1.0	
Yes	228 (68.3)	13 (50.0)	0.5 (0.2–1.0)	0.063
**Obtains syringes from questionable sources**				
No	257 (77.0)	19 (73.1)	1.0	
Yes	77 (23.1)	7 (26.9)	1.2 (0.5–3.0)	0.658
**Opposite-sex regular partners**				
No	105 (31.4)	8 (30.8)	1.0	
Yes	229 (68.6)	18 (69.2)	1.0 (0.4–2.4)	0.944
**Opposite-sex casual partners**				
No	208 (62.3)	18 (69.2)	1.0	
Yes	126 (37.7)	8 (30.8)	0.7 (0.3–1.7)	0.474
**Opposite-sex client partners**				
No	290 (86.8)	19 (73.1)	1.0	
Yes	44 (37.7)	7 (26.9)	2.4 (1.0–6.1)	0.076
**Regular same-sex partners**				
No	326 (97.6)	24 (92.3)	1.0	
Yes	8 (2.4)	2 (7.7)	3.4 (0.7–16.9)	0.183
**Casual same-sex partners**				
No	320 (95.8)	25 (96.2)	1.0	
Yes	14 (4.2)	1 (3.9)	0.9 (0.1–7.2)	0.932
**Client same-sex partners**				
No	329 (98.5)	23 (88.5)	1.0	
Yes	5 (1.5)	3 (11.5)	8.6 (1.9–38.2)	0.013
***Egocentric network variables (maximum number of network members = 20)***				
**Total number of IDU in network**				
Median (IQR)	3 (2,5)	2 (0,5)	0.9 (0.8–1.1)	0.241
**Total number of IDU family members in network**				
Median (IQR)	0 (0,1)	0 (0,2)	1.2 (0.9–1.6)	0.260
**Spouse IDU in network**				
No	215 (64.4))	17 (65.4)	1.0	
Yes	119 (35.6)	9 (34.6)	1.0 (0.4–2.2)	0.917
***IDU risk network variables (maximum number of IDU = 5)***				
**Number of IDU respondent sees daily**				
None	107 (32.0)	12 (46.2)	1.0	
1	112 (33.5)	6 (23.1)	0.5 (0.2–1.3)	0.154
2	58 (17.4)	5 (19.2)	0.8 (0.3–2.3)	0.637
3–5	57 (17.1)	3 (11.5)	0.5 (0.1–1.7)	0.256
**Number of IDU in network who inject daily**				
None	191 (57.2)	17 (65.4)	1.0	
1	63 (18.9)	2 (7.7)	0.4 (0.1–1.6)	0.176
2	45 (13.5)	4 (15.4)	1.0 (0.3–3.1)	0.998
3–5	35 (10.5)	3 (11.5)	1.0 (0.3–3.5)	0.954
**Number of IDU in network who have been IDU for >5 years**				
0	159 (47.6)	14 (53.9)	1.0	
1	65 (19.5)	3 (11.5)	0.5 (0.1–1.9)	0.323
2	52 (15.6)	4 (15.4)	0.9 (0.3–2.8)	0.819
3–5	58 (17.4)	5 (19.2)	1.0 (0.3–2.8)	0.969
**Number of IDU in network who have injected at a hotel**				
0	207 (62.0)	20 (76.9)	1.0	
1	64 (19.2)	2 (7.7)	0.3 (0.1–1.4)	0.135
2–5	63 (18.9)	4 (15.4)	0.7 (0.2–2.0)	0.458
**Number of IDU in network who have injected at a shooting gallery**				
0	254 (76.1)	20 (76.9)	1.0	
1	43 (12.9)	2 (7.7)	0.6 (0.1–2.6)	0.488
2–5	37 (11.1)	4 (15.4)	1.4 (0.4–4.2)	0.582
**Number of IDU in network with whom respondent has pooled money**				
0	94 (28.1)	12 (46.2)	1.0	
1	91 (27.3)	4 (15.4)	0.3 (0.1–1.1)	0.074
2–3	90 (27.0)	6 (23.1)	0.5 (0.2–1.5)	0.213
4–5	59 (17.7)	4 (15.4)	0.5 (0.2–1.7)	0.292
**Number of IDU in network who have used a syringe before the respondent used it**				
0	255 (76.4)	20 (76.9)	1.0	
1	56 (16.8)	4 (15.4)	0.9 (0.3–2.8)	0.869
2–5	23 (6.9)	2 (7.7)	1.1 (0.2–5.0)	0.894
**Number of IDU in network who have used other drug paraphernalia before respondent has used it**.				
0	201 (60.2)	17 (65.4)	1.0	
1	59 (17.7)	3 (11.5)	0.6 (0.2–2.1)	0.429
2–5	74 (22.2)	6 (23.1)	1.0 (0.4–2.5)	0.932
**Number of IDU in network who have initiated respondent to injection drug use**				
0	242 (72.5)	21 (80.8)	1.0	
1	77 (23.1)	5 (19.2)	0.7 (0.3–2.1)	0.563
2–5	15 (4.5)	0 (0.0)	Not defined	
**Number of IDU in network who have shown respondent how to inject drugs**				
0	229 (68.6)	19 (73.1)	1.0	
1	75 (22.5)	6 (23.1)	1.0 (0.4–2.5)	0.940
2–5	30 (9.0)	1 (3.9)	0.4 (0.1–3.1)	0.382
**Number of IDU in network who have injected respondent with drugs**				
0	161 (48.2)	12 (46.2)	1.0	
1	95 (28.4)	5 (19.2)	0.7 (0.2–2.1)	0.525
2–5	78 (23.4)	9 (34.6)	1.5 (0.6–3.8)	0.344
**Number of IDU in network who have used talwin/ritalin**				
0	239 (71.6)	17 (65.4)	1.0	
1–3	54 (16.2)	4 (15.4)	1.0 (0.3–3.2)	0.944
4–5	41 (12.3)	5 (19.2)	1.7 (0.6–4.9)	0.315
**Number of IDU in network who have used cocaine**				
0	210 (62.9)	18 (69.2)	1.0	
1	55 (16.5)	3 (11.5)	0.6 (0.2–2.2)	0.481
2–3	44 (13.2)	4 (15.4)	1.1 (0.3–3.3)	0.919
4–5	25 (7.5)	1 (3.9)	0.5 (0.1–3.6)	0.467
**Number of IDU in network who have used morphine**				
0	255 (76.4)	21 (80.8)	1.0	
1	46 (13.8)	4 (15.4)	1.1 (0.3–3.2)	0.924
2–5	33 (9.9)	1 (3.9)	0.4 (0.0–2.8)	0.336

**Table 4:** HCV multivariate adjusted OR and 95% CI.

Variable name	OR (95% CI)	p value
**Years of injection drug use ** Categories based on quartiles (Q1–Q4 below)		
Q2 vs. Q1	3.1 (1.5–6.2)	0.002
Q3 vs. Q1	10.8 (4.8–24.5)	<0.001
Q4 vs. Q1	10.3 (4.7–22.6)	<0.001
**injected at a shooting gallery**		
Yes vs. no	4.3 (1.8–10.5)	<0.001
**Ever used someone else's used syringe**	See table 5	
**Number of IDU in network who use talwin/ritalin**	See table 5	
**Number of IDU in network who have shown respondent how to inject drugs**	See table 5	

**Table 5:** Adjusted odds ratios and 95% CI for interactions between injection with a used syringe and the IDU risk network variables.

A)			
		**Reported injection with used syringe**	
		
		**Yes**	**No**

**Number of talwin/ritalin users in IDU risk network**	0	7.9 (3.7–16.8) 95 HCV +/58 HCV - (62%)	1.00 24 HCV +/81 HCV - (22.9%)
	1–3	0.6 (0.3–42.4) 26 HCV +/10 HCV - (72%)	1.00 16 HCV +/7 HCV - (69.6%)
	4–5	1.0 (0.2–156.8) 28 HCV +/9 HCV - (76%)	1.00 9 HCV +/2 HCV - (81.8%)
B)			

		**Reported injection with used syringe**	
		
		Yes	No

**Number of IDU who have shown the respondent how to inject drugs**	0	7.9 (3.7–16.8) 111 HCV +/41 HCV - (73.0%)	1.00 36 HCV +/63 HCV - (36.4%)
	1	7.7 (0.3–37.1) 31 HCV +/23 HCV - (57.4%)	1.00 8 HCV +/20 HCV - (28.6%)
	2–5	0.4 (0.2–87.2) 7 HCV +/13 HCV - (33.3%)	1.00 5 HCV +/7 HCV - (41.6%)

(A) number of IDU in the network who used talwin/ritalin or (B) have shown the respondent how to inject. Serostatus data for HCV corresponding to each cell is also shown.

**Table 6:** HBV multivariate adjusted OR

Variable name	OR (95% CI)	p value
**Years of injection drug use ** Categories based on quartiles (Q1–Q4 below)		
Q2 vs. Q1	4.7 (1.6–13.9)	0.005
Q3 vs. Q1	11.3 (3.8–33.2)	<0.001
Q4 vs. Q1	15.7 (5.3–46.5)	<0.001
**Ever used someone else's used syringe**		
Yes vs. No	2.2 (1.2–4.0)	0.005
**injected at a shooting gallery**		
Yes vs. no	2.9 (1.3–6.3)	0.020
**Opposite-sex casual sex partners**		
Yes vs No	0.5 (0.3–0.9)	0.020
**Mobility (Moved to Winnipeg in past year)**		
Yes vs. no	0.3 (0.2–0.7)	0.004
**Number of IDU in network who use talwin/ritalin**		
1–3 vs. none	2.9 (1.4–5.9)	<0.005
4–5 vs none	3.2 (1.4–7.2)	<0.009
**Number of IDU in network who have intiated respondent to injection drug use**		
1 vs. none	0.6 (0.3–1.4)	0.248
2–5 vs. none	0.1 (0.02–0.8)	0.023
**Number of IDU in risk network who have injected at a hotel**		
1 vs. none	1.3 (0.6–2.6)	0.490
2–5 vs. none	2.8 (1.3–5.9)	0.006

**Table 7:** HIV multivariate adjusted OR

Variable name	OR (95% CI)	p value
**Ethnicity**		
Aboriginal vs. caucasian	4.4 (1.2–15.6)	0.022
**Ever used someone else's used syringe**		
Yes vs. No	8.7 (2.0–38.5)	0.004
**injected at a shooting gallery**		
Yes vs. no	3.0 (1.2–7.5)	0.018
**Same-sex client sex partners**		
Yes vs No	6.9 (1.5–32.4)	0.014
